# Excessive Iron Induces Macrophage Dysfunction in the Liver, Causing Adverse Pregnancy Outcomes in Mice

**DOI:** 10.3390/metabo15070431

**Published:** 2025-06-24

**Authors:** Sayaka Shimazaki, Ren Ozawa, Akari Isobe, Sohei Kuribayashi, Hisataka Iwata, Koumei Shirasuna

**Affiliations:** Department of Animal Science, Tokyo University of Agriculture, Atsugi 243-0034, Japanh1iwata@nodai.ac.jp (H.I.)

**Keywords:** iron, placenta, pregnancy complication, inflammation, macrophages, liver

## Abstract

Background: Iron is an important micronutrient under physiological conditions, including pregnancy. On the other hand, excessive iron intake is also associated with adverse pregnancy outcomes. Macrophages are crucial in regulating iron homeostasis and pregnancy conditions. However, the role of macrophages in iron metabolism during pregnancy is unclear. Therefore, we used mouse models to investigate whether maternal iron overload induces pregnancy complications and their interactions with macrophages. Methods and Results: Administration of high-dose iron (iron dextran) by intraperitoneal injection to pregnant mice induced pregnancy complications such as fetal death, but low-dose iron did not affect fetal weight. In the placenta, the amount of iron was significantly increased and levels of macrophages were decreased by iron administration. In the liver, iron administration dramatically increased the amount of iron, with increased inflammatory cytokines tumor necrosis factor-α (TNFα) and interleukin-6. Macrophages were observed to surround deposited iron in the liver. In an in vitro experiment, treatment with iron stimulated TNFα secretion with cell death in macrophages, but not in liver cells. To investigate the importance of macrophages during pregnancy, clodronate liposomes were administered to reduce macrophages in pregnant mice. The macrophage reduction in pregnant mice resulted in an increased absorption rate and fetal growth restriction, together with higher iron accumulation and inflammatory cytokines in the liver. Conclusions: Maternal excess iron may induce inflammatory conditions with macrophage dysfunction in the liver, resulting in pregnancy complications. The reduction in macrophages also induced higher iron levels and adverse effects during pregnancy, suggesting a vicious cycle between excessive iron and macrophage dysfunction during pregnancy.

## 1. Introduction

Iron is an important micronutrient metal in the body that is required by all tissues for cellular processes, such as DNA synthesis, ATP production in mitochondria, and heme [1]. Under physiological conditions, iron levels are tightly controlled by both dietary iron absorption and iron mobilization from liver stores. On the other hand, excessive iron can be toxic to tissues in the body. Under iron excessive conditions, iron accumulates in various tissues and induces cellular damage through the generation of reactive oxygen species [2]. As well, excess cellular iron contributes to iron-dependent programmed cell death, known as ferroptosis [3]. It has been reported that higher iron concentrations are associated with various diseases, such as obesity, liver dysfunction, and atherosclerosis [4,5,6,7].

Iron requirements increase substantially over the course of pregnancy to support fetal and placental growth and maternal erythropoietic expansion [8]. Therefore, iron deficiency is a severe risk factor for both mother and fetus, which has led to the therapeutic use of iron supplementation, even without screening for pre-existing iron deficiency [9]. Although iron supplementation is essential for pregnant women with iron deficiency, most women of reproductive age have adequate iron stores in the body; less than 25% of pregnant women in developed countries have mild iron deficiency and anemia [10]. High-iron status in pregnant women is associated with gestational diabetes, fetal growth restriction, preterm birth, and preeclampsia [11,12,13,14]. Recently, Fisher et al. [9] demonstrated that higher maternal iron levels potentiate embryonic death in the uterus via induction of the tumor necrosis factor-α (TNFα)-apoptosis axis during both acute inflammatory and in obese situations. The study highlighted the safety of indiscriminate iron supplementation during pregnancy and indicated the need for further investigation of the detailed mechanisms of iron toxicity during pregnancy [9].

Macrophages are crucial cells in the regulation of systemic iron homeostasis. Macrophages phagocytize and degrade damaged or senescent erythrocytes and recycle heme-associated iron, which can be stored as ferritin within macrophages or released into the plasma via the iron export protein ferroportin [15]. In addition, iron overloading regulates the macrophage phenotype toward a proinflammatory type (with an increase in inflammatory cytokine production such as TNFα), suggesting a role for iron metabolism in macrophage functioning in sterile inflammation [4,16]. Macrophages are also important during pregnancy. For example, macrophages stimulate progesterone synthesis and secretion in the ovaries by inducing angiogenesis within the corpus luteum, which results in the establishment of implantation [17]. Moreover, macrophages also have roles in the maintenance of pregnancy and parturition [18,19]. However, the role of macrophages in iron metabolism during pregnancy remains unclear.

Therefore, the present study aimed to clarify whether excessive iron is associated with pregnancy complications and whether macrophages play a role in iron metabolism during pregnancy using a mouse model.

## 2. Materials and Methods

### 2.1. Animals

All experiments were approved by the Tokyo University of Agriculture Animal Care and Use Committee and were carried out in accordance with the Guide for Care and Use of Laboratory Animals (No. 2022097). The ICR male and female mice were purchased from Japan SLC Inc. (Shizuoka, Japan). The mice were maintained on a 12 h light–dark schedule in an environment controlled for temperature (22–25 °C) and humidity. The mice received a standard diet (CE-2, 25.1% crude protein, 4.8% crude fat, 31.5 mg iron and 342.4 kcal in 100 g) purchased from CLEA Japan, Inc. (Tokyo, Japan), during the experiment.

### 2.2. Maternal Iron Overload Experimental Model

Female mice (10–15 weeks) were mated with male mice. The presence of a vaginal plug was confirmed the next morning, which was designated as the first gestational day (GD1). To evaluate the effect of acute iron loading, mice randomly received an intraperitoneal (i.p.) injection of iron dextran (Sigma-Aldrich, St. Louis, MO, USA) as reference [4]. Moon et al. [20] used three different doses of iron dextran (100, 200, and 300 mg i.p./mouse) and determined that administration of iron dextran (100 mg i.p./mouse) adequately increased plasma iron levels. In our preliminary experiment investigating with lower doses than those used by Moon et al. [20], an i.p. injection of iron dextran was administered for three consecutive days at doses of 100, 500, or 1000 mg/kg of body weight (approximately 12–120 mg/mouse) in non-pregnant female mice. Although body weight did not change during the experiment, the plasma iron concentration was increased by iron injection at doses of 500 and 1000 mg/kg of body weight. Therefore, considering the maternal weight gain during pregnancy, iron dextran was administered twice to pregnant mice, on GD8 and GD12, at a concentration of 400 (n = 8) or 800 (n = 6) mg/kg of body weight in order to examine the effect on the time of placental formation after implantation. The same volume of saline was administered to pregnant mice in the control group (n = 6). The body weights of pregnant mice were measured on GD1, GD8, GD12, and GD17. At GD17, blood pressure was measured by the tail-cuff method (BP-98A; Softron Ltd., Tokyo, Japan), as previously described [21,22]. On GD17, pregnant mice were anesthetized with isoflurane and killed by exsanguination; the uteri were opened and whole placental tissues were collected after counting the number of fetuses and weighing them [21]. The weights of placentas and fetuses were averaged for all animals. Blood was collected from the abdominal vein using a heparinized 1 mL syringe and a 26 G needle. The blood was centrifuged at 2500 rpm at 4 °C to obtain plasma components, which were then stored at −20 °C until analysis. The liver was collected and weighed. The liver and placentae without solution were stored at −80 °C until analysis. Although no pregnancy abnormalities were observed in the control and 400 mg/kg iron dextran groups, the 800 mg/kg iron dextran group showed a higher ratio of pregnancy complications. Therefore, because it was difficult to analyze the effects on tissues in the 800 mg/kg iron dextran group, we decided to proceed with the analysis using the 400 mg/kg iron dextran group.

### 2.3. Effects of Maternal Iron Overload on Inflammatory Cytokines in the Liver and Placenta

To investigate the effects of iron overload during pregnancy on the secretion of inflammatory cytokines, liver and placental tissues were cultured as previously described [23]. Briefly, the liver and placental tissues of the mother in both the control and iron overload groups (400 mg/kg i.p.) were cut into small pieces (approximately 50 mg and 20 mg wet weight of liver and placenta, respectively) and placed in 24-well plates (Thermo Fisher Scientific, Inc., Waltham, MA, USA) containing 1 mL of Dulbecco’s modified Eagle’s medium/F-12 (DMEM/F-12; Life Technologies Corporation, Carlsbad, CA, USA) supplemented with amphotericin B, gentamicin (Sigma-Aldrich), and 5% fetal calf serum (FCS, ICN Pharmaceuticals, Inc., Costa Mesa, CA, USA) for 24 h at 37 °C. The supernatants were collected and stored at −20 °C prior to ELISA. The analyzed groups were control liver (n = 3), iron overload liver (n = 4), control placenta (n = 5), and iron overload placenta (n = 5), and experiments were performed with n = 3–4 per individual.

### 2.4. Measurements of Iron, Ferritin, and Inflammatory Cytokines

Iron concentrations in the blood plasma and tissue homogenates were determined using the metalloassay iron detection kit (FE31M, Metallogenics Co., Ltd., Chiba, Japan) according to the manufacturer’s instructions. In the placental tissues, data were obtained as two biological replicates in individuals. Ferritin concentrations in the blood plasma were determined using a mouse ELISA kit (E-90F, Immunology Consultants Laboratory, Portland, OR, USA) according to the manufacturer’s instructions. The levels of TNFα, interleukin (IL)-6, and soluble fms-like tyrosine kinase (sFlt-1) in the tissue culture supernatants were measured using a mouse ELISA kit (DY410, DY406, and DY471, R&D system, Minneapolis, MN, USA) according to the manufacturer’s instructions. The absorbance at 450 nm was measured using a microplate spectrophotometer (Bio Tec Epock, DS Pharma Biomedical, Osaka, Japan).

### 2.5. Histology of the Liver and Placental Tissue

For histological analysis, liver and placental tissues were fixed in 4% paraformaldehyde (FUJIFILM Wako Pure Chemical Corporation, Osaka, Japan) and embedded in paraffin. Paraffin sections (5 µm in thickness) were cut using a microtome. The sections were de-waxed and stained with hematoxylin and eosin (H-E staining, Genostaff Co., Ltd., Tokyo, Japan). To examine iron deposition, the sections were stained Berlin blue (Genostaff Co., Ltd.). Immunohistochemical staining for F4/80 (macrophage marker, anti-F4/80 rat monoclonal antibody, MCA497, 1 μg/mL, Bio-Rad Laboratories Inc., Hercules, CA, USA) was performed with DAB or chromogenic substrate (Leica Green DC9913, Leica Biosystems, Nussloch, DE, Germany) using manufactural protocol (Genostaff Co., Ltd.). For negative control staining, normal rat IgG2b (MAB0061, R&D systems) was used. Sections were photographed using the NanoZoomer S210 virtual slide scanner (Hamamatsu Photonics, Shizuoka, Japan).

### 2.6. Real-Time qPCR

After collecting the liver and placenta, total RNA was prepared using ISOGEN II (Nippon Gene Co., Ltd., Tokyo, Japan) according to the manufacturer’s instructions, and cDNA production was performed using a commercial kit (ReverTra Ace; Toyobo, Tokyo, Japan). Real-time qPCR was performed using CFX Connect^TM^ Real Time PCR (Bio-Rad Laboratories Inc.) to detect the mRNA expression of *EGF-like module-containing mucin-like hormone receptor-like 1 (Emr1)* and *glyceraldehyde 3-phosphate dehydrogenase* (*Gapdh*). The antisense and sense primers used were *Emr-1* (5’-CCTGGACGAATCCTGTGAAG-3′ and 5′-GGTGGGACCACAGAGAGTTG-3′) and *Gapdh* (5′-TGTGTCCGTCGTGGATCTGA-3′ and 5′-TTGCTGTTGAAGTCGCAGGAG-3′). RT-qPCR was performed using SYBR green (Thunderbird SYBR qPCR Mix, Toyobo). The expression level of each target gene was normalized to the corresponding *Gapdh* threshold cycle values using the ΔΔ CT comparative method [24].

### 2.7. Cell Culture and in Vitro Experiments

Murine macrophage cell line (J774) were cultured in DMEM/F-12 medium supplemented with antibiotics (amphotericin B and gentamicin) and 10% FCS. J774 cells were seeded at a concentration of 4 × 10^5^ cells/well in 48-well culture plates (Thermo Fisher Scientific). The murine liver cell line (NCTC clone 1469, JCRB9075 [25]) was purchased from the National Institutes of Biomedical Innovation. Briefly, NCTCs were cultured in DMEM/F-12 supplemented with antibiotics and 10% FCS. Then, NCTCs were seeded at a concentration of 5 × 10^4^ cells/well in 48-well culture plates.

To investigate the direct role of iron in these cells, iron (II) sulfate (FeSO_4_), at a concentration of 10 or 100 μM (Sigma-Aldrich, as a reference for treatment concentration [26]), was added for 24 h and the supernatants were collected. The supernatants were collected and stored at −20 °C prior to ELISA. Data were obtained from at least two independent experiments as technical replicates (n = 3–4 in each group/experiment as a biological replicate).

### 2.8. Determination of Lactate Dehydrogenase (LDH)

The levels of LDH, a cell death marker, in cell culture supernatants were determined using a cytotoxicity LDH assay kit (DOJINDO Laboratories, Kumamoto, Japan). The absorbance at 490 nm was measured using a microplate spectrophotometer (Bio Tec Epock, DS Pharma Biomedical).

### 2.9. Determination of Iron Uptake

We examined the iron uptake capacity of cells. Cells (J774 and NCTC cells) were incubated with or without iron (II) sulfate for 24 h and then further incubated with a reagent for measuring of intracellular iron ion levels (Ferro Orange, 1 μM for 30 min, DOJINDO Laboratories). Intracellular iron ion levels were determined using a fluorescence microplate reader (Spark, Tecan Group, Tokyo, Japan) according to the manufacturer’s instructions.

### 2.10. Maternal Macrophage Reduction Experimental Model

To evaluate the role of macrophages, mice received an i.p. injection of clodronate or control liposomes (Katayama Chemical Industries, Osaka, Japan) as a reference [4]. In our preliminary experiment, clodronate or control liposomes were administered twice at 4-day intervals at doses of 25 or 37.5 mg/kg of body weight to non-pregnant female mice. Although body weight did not change during the experiment, the weights of the spleen and liver were decreased by clodronate liposome injection at a dose of 37.5 mg/kg of body weight. Therefore, clodronate liposomes were administered twice to pregnant mice at 25 mg/kg of body weight on GD8 and GD12 (n = 11). The same dose of control liposomes was administered to pregnant mice in the control group (n = 6). The body weights of pregnant mice were measured on GD1, GD8, GD10, GD12, GD14, and GD17. On GD17, pregnant mice were sacrificed, uteri were opened, and whole placental tissues were collected after counting the number of fetuses and weighing them. When no clear absorbed fetus was observed, the number of implantation sites (black trace in the endometrium) was counted. The fetal absorption rate was calculated using the following equation: fetal absorption rate = (the number of absorbed fetuses)/(the number of viable fetuses + absorbed fetuses with implantation sites) [21]. Blood was collected from the abdominal vein using a heparinized 1 mL syringe and a 26 G needle. The blood was centrifuged at 2500 rpm at 4 °C to obtain plasma components, which were then stored at −20 °C until analysis. The liver was collected and weighed. The liver and placentae without solution were stored at −80 °C until analysis.

### 2.11. Statistics

Data are expressed as mean ± SEM, as indicated in the figure legends. Differences between the treatment groups were identified using the Mann–Whitney U test. Multiple comparisons were made using the Kruskal–Wallis test with Steel’s comparison test using statistical software (BellCurve version 4.02, Social Survey Research Information, Tokyo, Japan). Statistical significance was set at *p* < 0.05.

## 3. Results

### 3.1. Effects of Iron Overload in Pregnant Mice

**Figure 1:**
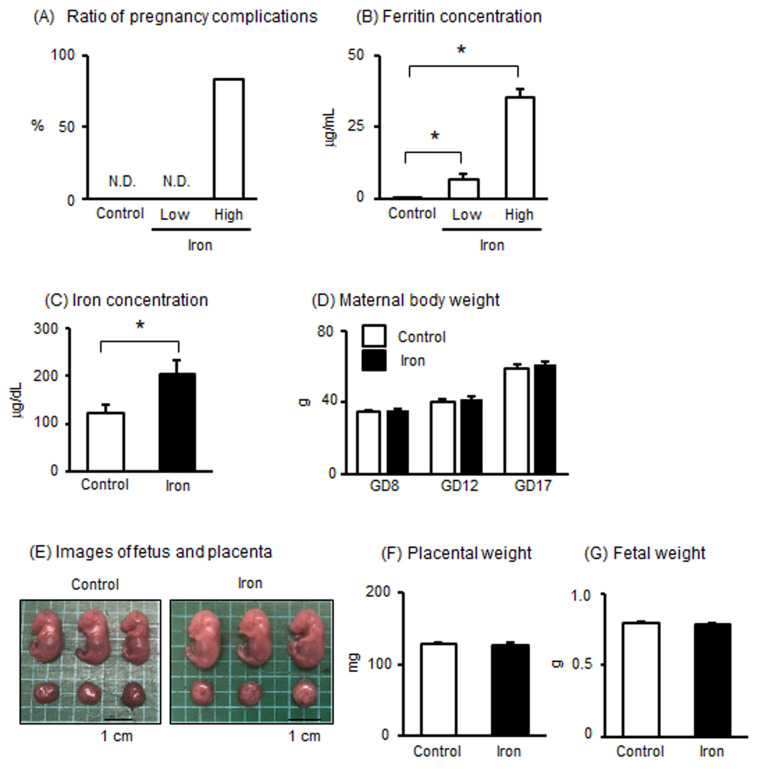
Effects of iron overload in pregnant mice. (**A**) Effects of iron administration at low and high doses (400 mg/kg, n = 8; 800 mg/kg, n = 6) with respect to the occurrence of pregnancy complications compared with the control group (saline administration, n = 6). (**B**) Ferritin concentration in the peripheral plasma after saline or iron administration. (**C**) Iron concentration in the peripheral plasma after saline or iron administration at low dose. (**D**) Maternal body weight at GD8, 12, and 17 after iron (low dose) or saline administration. (**E**) Typical images of fetus and placenta. (**F**,**G**) Weight of placenta and fetus at GD17. Data are expressed as mean ± SEM. * *p* < 0.05.

To test our hypothesis that excessive iron is associated with pregnancy dysfunction in the entire maternal body, we investigated the effects of iron overload in pregnant mice. Considering the preliminary experiment, iron dextran was administered to pregnant mice at a concentration of 400 (low dose) or 800 (high dose) mg/kg of body weight at GD8 and GD12. While no pregnancy abnormalities were observed in the control and low-dose iron groups, high-dose iron administration significantly induced pregnancy complications in 83.3% of the six dams, with three maternal deaths and two abortions (Figure 1A). Generally, ferritin levels in circulation are included in the diagnostic criteria for iron overload. We showed that plasma ferritin levels significantly increased after administration of iron (400 and 800 mg/kg, Figure 1B), confirming an iron overload situation in this experiment. These findings indicate that pregnancy complications were induced by high-dose iron in mice.

Since it was difficult to analyze the effects on tissues in the high-dose iron-administered group (800 mg/kg iron), we decided to proceed with the analysis using low-dose iron administration (400 mg/kg iron). Determinations of plasma iron concentrations confirmed higher levels of iron concentration after administration of iron (400 mg/kg) in pregnant mice (Figure 1C). No differences were evident in maternal body weight from GD8 to GD17 between the control and iron-administered groups (Figure 1D). There were no differences in litter size (14.0 ± 0.6 in the control group; 13.6 ± 0.6 in the 400 mg/kg-administered group). Typical fetal and placental images are shown in Figure 1E. There were no differences in placental and fetal weight (Figure 1F,G).

### 3.2. Effects of Iron Overload in Placenta of Pregnant Mice

**Figure 2:**
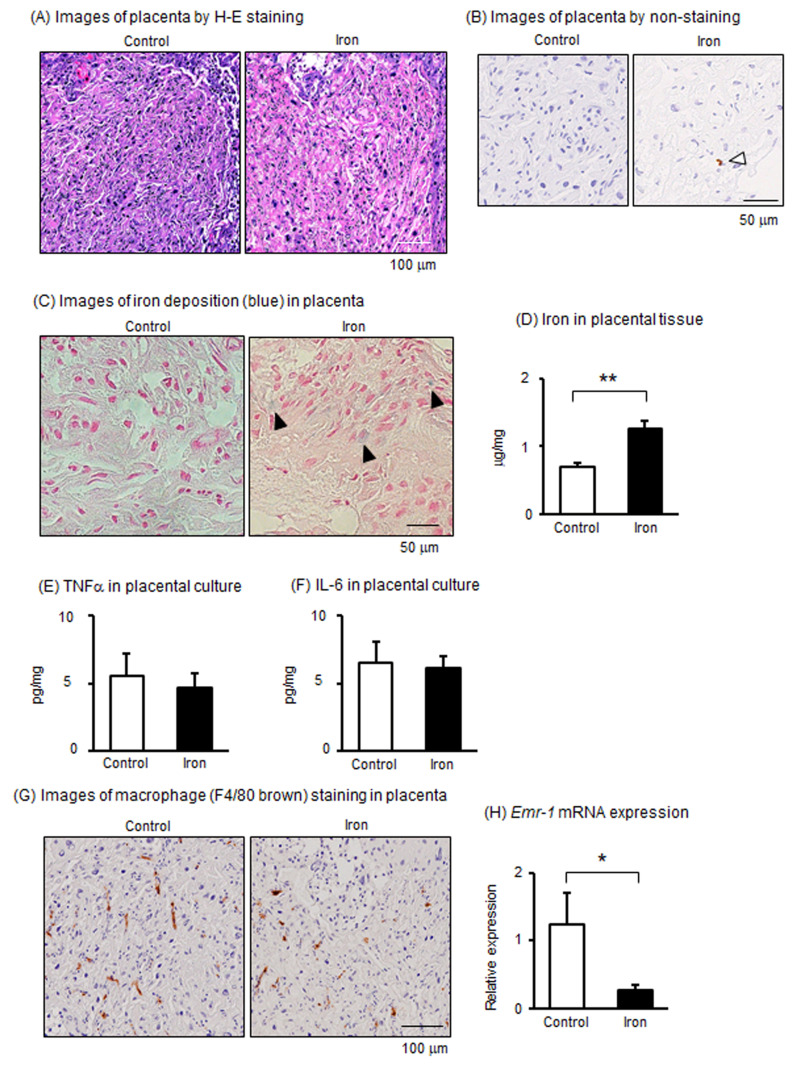
Effects of iron overload on placentas in pregnant mice. (**A**) Images of placentas by H-E staining. (**B**) Images of placentas with non-H-E staining. White arrows indicate areas of iron deposition (brown color). (**C**) Images of iron deposition (blue color) in placentas. Black arrows indicate areas of iron deposition. (**D**) Iron concentration in the placenta. (**E**,**F**) TNFα and IL-6 levels in supernatants of placental tissue cultured for 24 h. (**G**) Images of placental macrophages stained by F4/80 (brown-colored cells). (**H**) mRNA levels of *Emr-1* in placental tissues. Data are expressed as mean ± SEM. * *p* < 0.05 or ** *p* < 0.01.

Next, we examined the effects of iron overload on placental function. In histological analysis using H-E staining, no effects from iron administration were observed on placental structure (Figure 2A). On the other hand, in the non-staining histological section, brown-stained deposits were observed in the placenta after administration of iron (Figure 2B). Therefore, we examined iron deposition in the placenta with Berlin blue; although not many, iron deposits were observed in the placenta (Figure 2C). Determination of iron concentration in the placental tissues confirmed an increase in iron concentration in the iron-administered group, indicating an iron-overload condition in the placenta (Figure 2D).

Iron overload has been reportedly associated with inflammation with macrophages [9]. Therefore, major inflammatory cytokines, including TNFα and IL-6, were assessed. However, in the supernatants of the placenta, the secretion of TNFα and IL-6 did not differ between the control and iron-administered groups (Figure 2E,F). On the other hand, F4/80, known as Emr-1, is a protein encoded by the *Adgre1* gene and well known as a marker of macrophages. Although many F4/80-positive macrophages (brown-colored positive cells) were mainly localized in the labyrinth area of the placenta, a slight decrease in macrophages was observed in the placentas of the iron-administered group (Figure 2G). Indeed, mRNA expression of *Emr-1* significantly decreased in the placentas of the iron-administered group in comparison to those in the control group (Figure 2H). These results indicated that when iron is administered at a concentration that does not have a significant effect on pregnancy, it accumulates in the placenta and affects macrophages, but does not increase the production of inflammatory cytokines.

### 3.3. Effects of Iron Overload in the Liver of Pregnant Mice

**Figure 3:**
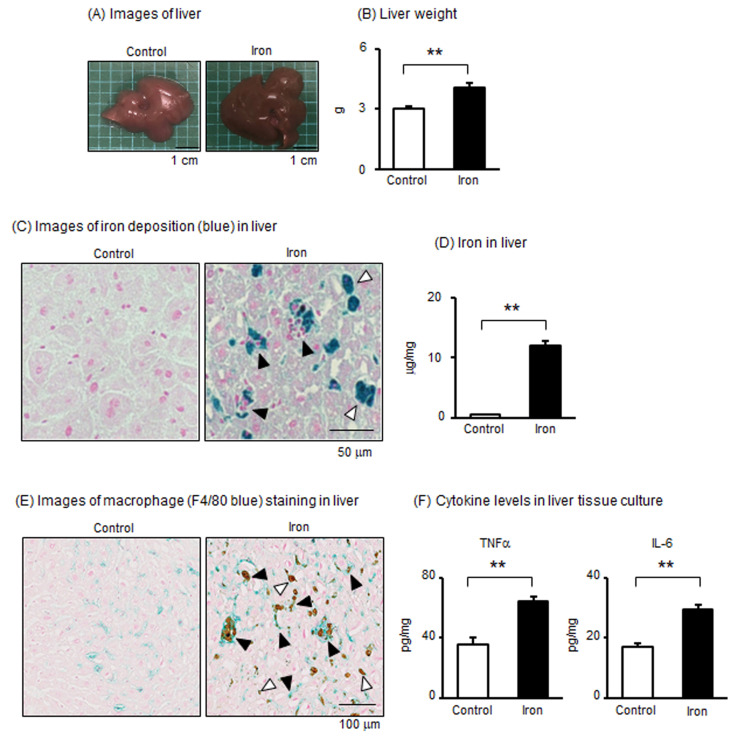
Effects of iron overload on the liver in pregnant mice. (**A**) Typical images of the liver. (**B**) Weight of the liver at GD17. (**C**) Images of iron deposition (blue color) in the liver. White arrows indicate the area of iron deposition (blue color) in large liver cells and black arrows indicate the area of iron deposition with small cell accumulation. (**D**) Iron concentration in the liver. (**E**) Images of macrophages in the liver stained by F4/80 (blue-colored cells). Black arrows indicate the area of iron deposition (brown color) surrounding macrophages and white arrows indicate the area of iron deposition without macrophages. (**F**) TNFα and IL-6 levels in supernatants of liver tissue cultured for 24 h. Data are expressed as mean ± SEM. ** *p* < 0.01.

The liver is important for iron metabolism. Typical images of the liver are shown in Figure 3A; the administration of iron causes the liver to turn black. Liver weight was significantly increased by iron administration compared with that in the control group (Figure 3B). We further confirmed iron deposition in the liver of the iron-administered group by Berlin blue staining (Figure 3C). In addition, the amount of iron in the liver was significantly higher in the iron-administered group than in the control group (Figure 3D). In the liver, many F4/80-positive macrophages (blue-colored positive cells) were observed in the control group (Figure 3E). Similar to the placenta, brown-stained areas were observed in the liver of the iron-administered group, suggesting the deposition of iron (Figure 3E and Figure S1A,B). Interestingly, many F4/80-positive macrophages (blue-colored positive cells) were observed around the iron deposits (black arrowhead, Figure 3E). It was also observed that macrophages had not yet accumulated around the iron (white arrowhead, Figure 3E). Areas where large cells were strongly stained (white arrowhead) and areas where small cells were accumulated in the stained areas (black arrowhead) can be observed in Figure 3C, suggesting that iron is accumulated in liver parenchymal cells and macrophages, respectively. Then, to investigate the effect of iron accumulation in tissues, liver tissues were cultured. As expected, the secretion of TNFα and IL-6 was significantly higher in the tissue culture supernatants of the liver in the iron overload group than in the control group (Figure 3F). Therefore, it is possible that iron administration affected macrophages and parenchymal cells in the liver of pregnant mice, inducing the production of pro-inflammatory cytokines.

### 3.4. Effects of Iron Overload on Blood Pressure and Anti-Angiogenic Factors in Pregnant Mice

It has been reported that iron levels are higher in preeclamptic patients than in healthy pregnant women, suggesting an association between higher iron levels and the pathophysiology of preeclampsia [27]. Therefore, we examined the levels of blood pressure and preeclampsia-associated anti-angiogenic factors, such as sFlt-1 (a soluble receptor for vascular endothelial growth factor). sFlt-1 is mainly derived from the placenta and contributes to the pathogenesis of preeclampsia [28,29,30,31]. However, iron administration had no effect on either systolic or diastolic blood pressure in pregnant mice (Figure S2A). Also, in blood plasma and placental tissue cultures, the level of sFlt-1 did not differ between the control and iron-administered groups (Figure S2B,C). These findings suggest that this experimental model of iron overload during pregnancy in mice is not associated with the pathogenesis of preeclampsia.

### 3.5. Effects of Iron on Macrophages and Liver Cells

**Figure 4:**
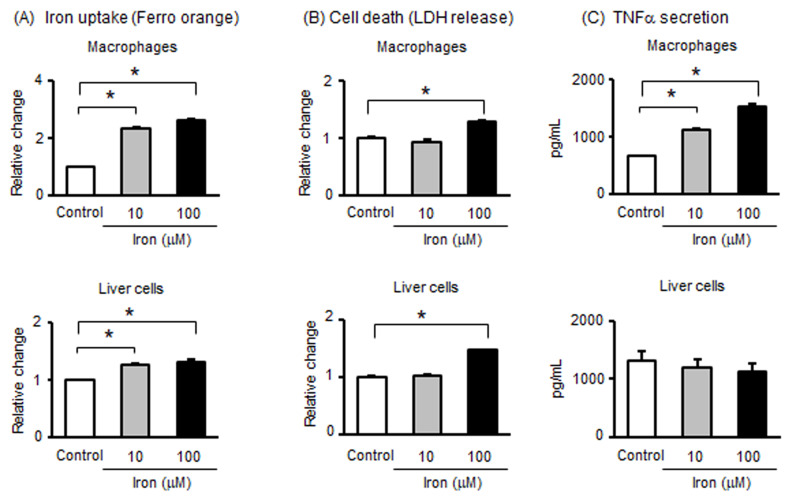
Effects of iron in macrophages and liver cells. Mouse macrophage and liver cell lines were incubated for 24 h in the presence or absence of iron. (**A**) After incubation, levels of intracellular iron were measured. (**B**) After incubation, LDH in the supernatant was determined. (**C**) After incubation, TNFα concentrations in the supernatant were determined using ELISA. Data are expressed as mean ± SEM. * *p* < 0.05.

Using cell culture models, we investigated the effects of iron overload. Therefore, macrophages (J774, mouse macrophage cell line) and liver cells (mouse NCTC liver cell line) were treated with iron for 24 h. At first, we evaluated iron uptake in macrophages and liver cells. Iron was obviously taken up by macrophages and slightly (but significantly) taken up by liver cells (Figure 4A). Then, treatment with iron significantly increased LDH release in macrophages and liver cells (Figure 4B). Considering the uptake of iron by macrophages and liver cells, we examined the effect of iron on TNFα secretion. Treatment with iron significantly stimulated TNFα secretion from macrophages, but not from liver cells (Figure 4C). These results indicate that macrophages, not liver cells, take up iron within the cells, resulting in the secretion of TNFα with cell death.

### 3.6. Effects of Macrophage Reduction in Pregnant Mice

**Figure 5:**
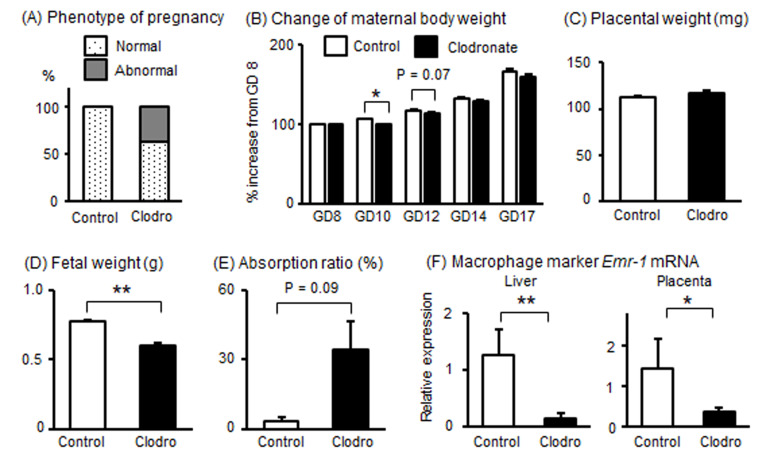
Effects of macrophage reduction in pregnant mice. Clodronate liposomes (n = 11) or control liposomes (n = 6) were administered twice to pregnant mice on GD8 and GD12. (**A**) Ratio of phenotype of pregnancy at GD17. (**B**) Relative change in maternal body weight from GD8 to GD17. (**C**,**D**) Weight of placenta and fetus at GD17. (**E**) Absorption ratio at GD17. (**F**) mRNA levels of *Emr-1* in liver and placental tissues. Data are expressed as mean ± SEM. * *p* < 0.05 or ** *p* < 0.01.

Next, to clarify the importance of macrophages during iron overload with respect to the induction of adverse pregnancy outcomes in mice, clodronate or control liposomes were injected into pregnant mice to reduce macrophages. When clodronate liposomes were administered to 11 pregnant mice, pregnancy continued in seven of the mice until GD17. The remaining four mice developed abnormal pregnancies (36.4%, Figure 5A), which included the death of three pregnant mice and a preterm birth by one pregnant mouse. As shown in Figure 5B, maternal body weight during GD10-12 decreased in the clodronate-injected group (n = 7, continued pregnancy) compared to that in the control liposome-injected group (n = 6). In the mice where pregnancy continued, placental weight did not change and fetal weight was significantly lower in the clodronate-injected group than in the control group (Figure 5C,D). The absorption rate of the embryo tended to be higher in the clodronate-injected group (Figure 5E). Confirming the effect of clodronate liposome injection, mRNA expression of *Emr-1* was significantly reduced by the injection of clodronate liposomes in both the liver and placenta (Figure 5F), suggesting that macrophages were reduced by clodronate liposomes in pregnant mice. These findings indicated that macrophage reduction in pregnant mice is associated with abnormal pregnancy outcomes.

### 3.7. Effects of Macrophage Reduction in the Liver of Pregnant Mice

**Figure 6:**
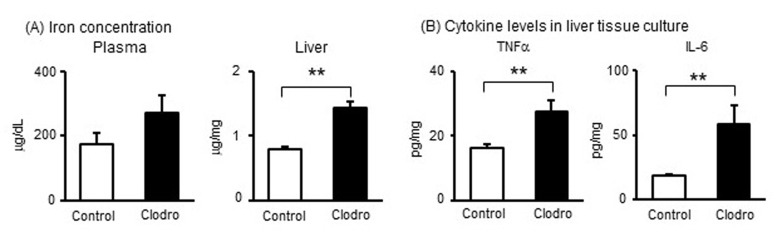
Effects of macrophage reduction in pregnant mice on the liver and placenta. Pregnant mice were administered clodronate liposomes or control liposomes twice (on GD8 and GD12). (**A**) Iron concentration in the plasma and liver. (**B**) TNFα and IL-6 secretion levels in supernatants of liver tissue cultured for 24 h. Data are expressed as mean ± SEM. ** *p* < 0.01.

Figure 3 shows the correlation between reduction in macrophages via iron overload and increased production of inflammatory cytokines in pregnant mice. Therefore, we examined the effects of macrophage reduction on levels of iron and cytokine concentrations. Although iron concentrations in plasma were slightly increased after administration of clodronate, iron concentrations in liver tissues significantly increased after administration of clodronate (Figure 6A), indicating an iron overload situation via macrophage reduction. In the tissue culture supernatants of the liver, secretion of TNFα and IL-6 was significantly higher in the clodronate-injected group than in the control group (Figure 6B). These findings suggest that macrophage reduction results in iron accumulation and induces inflammatory cytokine secretion in pregnant mice.

## 4. Discussion

The present results indicate that excessive iron during pregnancy directly affects the liver of the mother as well as the placenta via the accumulation of iron in these tissues. Iron accumulation results in the occurrence of pregnancy complications in mice, such as fetal death, preterm birth, and maternal death. Recently, Guo et al. [32] reported that high dietary iron supplementation during pregnancy clearly induced iron deposition in the maternal liver and placenta, with a positive correlation between placental iron concentration and offspring weight reduction in a mouse model. Similarly, Fisher et al. [9] showed that both genetic and dietary maternal iron overload results in higher levels of placental iron and embryonic iron stores. Moreover, the authors demonstrated that maternal exposure to both excess iron and inflammation causes embryo malformations and fetal death due to the induction of apoptosis in endothelial cells in the placenta and lung of the fetus [9]. These results suggest that excessive iron supplementation can have adverse effects on maternal conditions and fetal growth during pregnancy.

In the present study, the highest administered dose of iron resulted in adverse effects in pregnant mice, including a higher absorption rate with respect to the embryo and fetal growth restriction. Previously, Moroishi et al. [33] investigated the role of FBXL5 (F box and leucine-rich repeat protein 5), which is associated with iron metabolism. They showed that mice deficient in *Fbxl5* died in utero due to excessive iron accumulation [33]. Moreover, they also showed that liver-specific deletion of *Fbxl5* resulted in dysregulation of iron homeostasis, leading to the development of steatohepatitis, and that these mice died with acute liver failure when fed a high-iron diet [33]. Also, it has been reported that excessive iron supplementation in mice aggravates symptoms associated with liver inflammation, nonalcoholic fatty liver disease, and type 2 diabetes mellitus [5,6]. These collective findings suggest that excess iron accumulation is an indicator of the exacerbation of inflammation.

Generally, iron overload stimulates inflammatory cytokines from various types of cells and tissues, both in vivo and in vitro [4,8,26,34]. The present results also reveal higher levels in inflammatory cytokines (TNFα and IL-6) in the liver associated with higher iron deposition. Macrophages/Kupffer cells constitute the largest number of non-parenchymal cells in the liver and contribute to iron metabolism and other cellular functions in the liver [35]. Therefore, we hypothesized that macrophages/Kupffer cells participate in the increase in inflammatory cytokines following iron administration in the present study. Interestingly, in histological analysis, macrophages were observed to surround iron-deposited and iron-accumulated cells in the liver, suggesting the occurrence of a unique histological structure termed a “crown-like structure”, which are reported by Kanamori et al. [4] that macrophages/Kupffer cells surround dead hepatocytes and scavenge their debris in the liver of non-alcoholic steatohepatitis. Moreover, administration of iron resulted in phagocytosis of iron by macrophages with upregulation of inflammatory cytokines in the liver of mice fed a Western diet [4]. Therefore, we suggest that macrophages/Kupffer cells play a role in the elimination of iron-deposited cells, resulting in the induction of inflammation and cell death.

To determine the effects of iron deposition in the liver, iron was added to cells cultured in vitro. Interestingly, treatment with iron clearly induced LDH release both in macrophages and liver cells, but TNFα secretion was only increased by iron treatment in macrophages, suggesting that iron overload induces inflammation and subsequent cell death, especially in macrophages during pregnancy. Many studies have reported that iron-dependent cell death and inflammation are related to ferroptosis, necroptosis, pyroptosis, or apoptosis, with or without inflammatory cytokines, in various cell types [7,26,34,36]. Indeed, higher iron levels significantly stimulate inflammatory cytokines, such as TNFα and IL-1β, and cell death due to the induction of reactive oxygen species, lysosome damage, and mitochondrial dysfunction in various types of macrophages [5,34]. On the other hand, other types of iron (such as ferric citrate) significantly induced cell death via ferroptosis in primary hepatocytes [36]. Moreover, treatment with iron can also stimulate cytokine secretion with cell death in endothelial cells and vascular smooth muscle cells [7]. These results indicate that the effect of iron overload may depend on the type of cell.

In the present study, iron overload clearly affected macrophage/Kupffer cell function during pregnancy in mice. We next investigated whether macrophages contributed to the adverse effects of excessive iron or protected against these effects during pregnancy. Therefore, clodronate liposomes were administered to pregnant mice to transiently reduce the macrophage population in the body. Interestingly, macrophage reduction in pregnant mice resulted in an increase in absorption rate, fetal growth restriction, and maternal death, together with greater iron accumulation and greater levels of inflammatory cytokines in the liver. These findings indicated the occurrence of pregnancy complications via reduction with macrophages, similar to the results in pregnant mice with iron overload. The collective findings indicate a protective role of macrophages against adverse effects of excessive iron during pregnancy. Importantly, Wu et al. [35] recently reported that in an experimental model of hepatic ischemia/reperfusion injury, macrophages accumulate in the liver and further macrophage extracellular traps are induced, which lead to ferroptosis in hepatocytes. Furthermore, iron overload exacerbated ferroptosis and liver damage. In this experimental model, the authors showed that depletion of macrophages by clodronate liposomes further enhanced hepatic ischemia/reperfusion-induced ferroptosis, suggesting that macrophages protect against hepatic injury [35].

Macrophages have a broad range of functions not only for immunity but also for reproduction. For example, after parturition, macrophages accumulate in uterine tissues and clear senescent cells to prepare for the next pregnancy [37]. These events indicate the role of macrophages in the process of physiological uterine remodeling and pregnancy success [37]. Moreover, depletion of maternal macrophages is associated with preterm birth, neonatal death, and postnatal growth impairment [18]. In this abnormal model, transplantation of M2 macrophages (anti-inflammatory type) reduces inflammation in the uterus and fetus, prevents premature birth, and improves neonatal survival [18]. Therefore, the authors demonstrated a critical homeostatic regulatory role for macrophages during late pregnancy [18]. On the other hand, macrophage dysfunction contributes to various types of pregnancy complications [17,18,19], which are also associated with higher levels of iron in humans and mice [13,14]. Indeed, increased body iron content has been related to many diseases, including liver diseases, obesity, diabetes, and cardiovascular diseases [4,5,7]. Interestingly, conditional macrophage-specific deletion of the *Fth* gene (ferritin heavy chain, iron-storage protein) results in reduced iron content and inhibited development of obesity and diabetes with inflammatory cytokines [38]. Thus, macrophage iron regulation may play a pivotal role in the pathogenesis of diseases associated with sterile inflammation [38]. These findings suggest that macrophages are essential at all stages of pregnancy, and macrophage-specific reduction in iron may be a target for pregnancy or metabolic disorders through the inhibition of inflammation.

In the present study, plasma iron concentration was increased to approximately 200 μg/dL by administration of iron dextran during pregnancy in mice. However, blood iron level is generally >300 mg/dL in hemosiderosis patients, including patients with secondary iron overload (Hamilton, MSD MANUAL Professional Version, Secondary Iron Overload), much higher than the levels in our experiment. On the other hand, Moon et al. [20] established the mouse model of secondary iron overload by iron dextran injection, with the plasma iron concentrations of approximately 1000–14000 μg/dL (262 μg/dL in the control group). Therefore, it is believed that there is a species difference with respect to concentrations judged to be evidence of iron overload between humans and mice.

A limitation of this study is that the mechanism of cell death induced by excessive iron in vivo remains unclear. On the other hand, we were unable to clarify whether the increase in iron concentration was due to a decrease in macrophages/Kupffer cells or whether the increase in iron concentration resulted in a decrease in macrophages/Kupffer cells. In addition, we have to consider the possibility that the iron administration was due to acute inflammation and was not physiological. As well, we have to investigate the effect of dietary iron overload or the combined effects of iron overload and macrophage reduction during pregnancy. Further analysis will be required to understand the importance and significance of these results.

## 5. Conclusions

Maternal excess iron directly induces the adverse effects of inflammation with macrophage dysfunction during pregnancy, resulting in pregnancy complications. Moreover, a reduction in macrophages is associated with higher iron levels and adverse effects during pregnancy, suggesting a vicious cycle between excessive iron and macrophage dysfunction during pregnancy. To support these suggestions, further research is needed in the future to determine how iron overload and macrophage abnormalities induce abnormal pregnancies.

## Data Availability

The data presented in this study are available upon request from the corresponding author for clear reason.

## Supplementary Materials

The following supporting information can be downloaded at https://www.mdpi.com/article/10.3390/metabo15070431/s1: Figure S1: Effects of iron overload in the liver; Figure S2: Effects of iron overload on preeclamptic phenotypes in pregnant mice.

## Abbreviations

The following abbreviations are used in this manuscript:

TNFα	tumor necrosis factor-α
GD	gestational day
IL	interleukin
sFlt-1	soluble fms-like tyrosine kinase
Emr1	EGF-like module-containing mucin-like hormone receptor-like 1
Gapdh	glyceraldehyde 3-phosphate dehydrogenase
LDH	lactate dehydrogenase
FBXL5	F box and leucine-rich repeat protein 5
Fth	ferritin heavy chain
