# Supplementary material for: Excessive Iron Induces Macrophage Dysfunction in the Liver, Causing Adverse Pregnancy Outcomes in Mice
Source: Metabolites. 2025 Jun 24;15(7):431. doi: 10.3390/metabo15070431 (PMC12298340; doi:10.3390/metabo15070431)
Supplement: Supplementary file 1 [file metabolites-15-00431-s001.zip › metabolites-3682276-supplementary.pdf]

Article, Special Issue, Effects of Stress on Animal Metabolism

Excessive iron induces macrophage dysfunction in the liver, causing adverse pregnancy outcome in mice

Sayaka Shimazaki[1#], Ren Ozawa[1#], Akari Isobe[1], Sohei Kuribayashi[1], Hisataka Iwata[1], and Koumei Shirasuna[1*]

Sup Figure S1

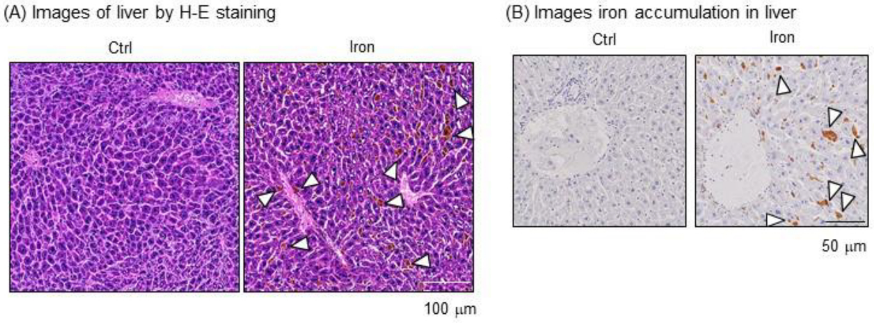

Supplementary Figure S1. Effect of iron overload in the liver
(A) Images of liver by H-E staining. White arrows indicate the area of iron deposition (blown color). (B) Images of liver with non-H-E staining. White arrows indicate the area of iron deposition (blown color).

Sup Figure S2

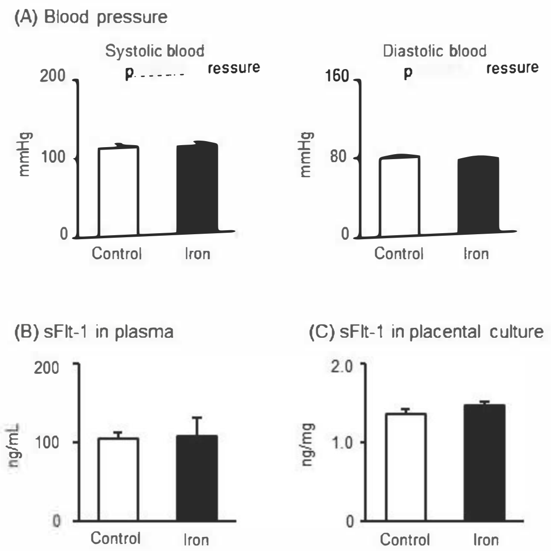

Supplementary Figure S2. Effect of iron overload on preeclamptic phenotypes in pregnant mice (A) Maternal systolic and diastolic blood pressure at GD17. (B and C) sFlt-1 levels in plasma and supernatants of placental tissue.
